# ERCC1 Single Nucleotide Polymorphism C8092A, but Not Its Expression Is Associated with Survival of Esophageal Squamous Cell Carcinoma Patients from Fujian Province, China

**DOI:** 10.1371/journal.pone.0106600

**Published:** 2014-09-05

**Authors:** Wan-Hua Chen, Pei-Ling Xin, Qun-Xiong Pan, Ya-Yun Chen, Cong-Ren Wang, Zhi-Shan Zhang, Yi-Feng Chen, Chao-Yang Zhang, Wen-Jie Cai

**Affiliations:** 1 Department of Clinical Laboratory, First Hospital of Quanzhou Affiliated to Fujian Medical University, Quanzhou, Fujian, People's Republic of China; 2 Department of Radiation Oncology, First Hospital of Quanzhou Affiliated to Fujian Medical University, Quanzhou, Fujian, People's Republic of China; 3 Department of Surgical Oncology, First Hospital of Quanzhou Affiliated to Fujian Medical University, Quanzhou, Fujian, People's Republic of China; 4 Department of Pathology, First Hospital of Quanzhou Affiliated to Fujian Medical University, Quanzhou, Fujian, People's Republic of China; National Cancer Institute, National Institutes of Health, United States of America

## Abstract

Esophageal carcinoma is one of the world's deadliest cancers. Esophageal squamous cell carcinoma (ESCC) is more frequent than adenocarcenoma (AC) in China. Platinum-based chemotherapy with surgical resection is a common treatment approach for ESCC; however, the treatment response is uncertain. Evidence suggests polymorphisms in genes encoding excision repair cross-complementing group 1 (ERCC1), a protein involved in nuclear excision repair (NER), may help predict response to cisplatin and other platinum-based chemotherapeutics. Multiple ERCC1 single nucleotide polymorphisms (SNPs) have been associated with platinum chemotherapy response. Two common SNPs occur at the C8092A and C118T loci. Our study aimed to determine if 1) an association exists between ERCC1 tumor expression and patient survival, 2) whether adjuvant therapy influence on survival is related to histological ERCC1 presence in tumor cell nuclei, and 3) whether other clinicopathological characteristics in a cohort of patients following surgery for various stages of ESCC are associated with tumor ERCC1 expression. One hundred eight patients were included in the study, and tumor biopsy was collected for genotyping and immunohistochemical analysis of ERCC1. Sixty-seven patients (62%) received no adjuvant therapy, and the rest had either platinum-based chemotherapy (28.5%), radiotherapy (6.5%) or both treatments (2.8%). Log-rank analysis revealed no significant connection between tumor ERCC1 expression (*P* = 0.12) or adjuvant therapy (*P* = 0.56) on patient survival. Also, non-parametric Mann-Whitney analysis showed no significant link between tumor size or nodus tumor formation and ERCC1 presence in patients in the study. Interestingly, C8092A SNP showed significant association with patient survival (*P* = 0.01), with patients homozygous for the mutant allele showing the most significantly reduced survival (*P* = 0.04) compared to those homozygous for the dominant allele (CC). Our results provide novel insight into the genotypic variation of patients from Quanzhou, Fujian province China.

## Introduction

Esophageal cancer, classified as adeno- (AC) or squamous cell carcinoma (SCC) is one of the top ten most malignant and deadly cancers worldwide. Of both forms of esophageal cancer, SCC is the most common globally and prognosis highly correlates with advancement or stage of the disease, and highly advanced cancers of the esophagus have poor prognostic outcomes. Combination of chemotherapy and adjuvant radiation treatment originally emphasized treatment of SCC [1]; however, meta-analysis of treatment effects versus tumor histopathology suggests this neo-adjuvant therapy provides similar benefits for AC and SCC, although clinical outcomes may benefit more in AC [2], In Westernized countries, esophageal AC is more common, although incidence is potentially declining. Conversely, Asian regions, such as rural and Northwest China, exhibit extremely high incidence of ESCC, especially in males. Despite debatable effects and clinical outcomes reported by analyses and randomized trials, many variables influence disease progression and prognosis which reflect the necessity for individualized approaches to cancer diagnosis and treatment.

Recently, much evidence suggests more severe and permanent outcomes from treatment effects involve damage or genome modifications of treated individuals. Currently, much research is targeting the identification of prognostic indicators for identification of presence or staging of specific cancers. A nucleotide excision repair (NER) protein known as the excision repair cross-complementing group 1 (ERCC1) may serve as a useful predictor for treatment response to cancer therapies. In general, single nucleotide polymorphisms (SNPs) have been associated to radiation and chemotherapy dependent pathways [3]. In non-small cell lung cancer (NSCLC), ERCC1 SNP has been linked to reduced survival time and increased risk of comorbid complications in specific populations of chemotherapy patients [4]. In particular, a documented association exists between ERCC1 mRNA levels and platinum and cisplatin therapies. Single nucleotide polymorphisms (SNP) of ERCC1 could affect potential for genetic repair, which highly influences the efficacy and individual sensitivity to these chemotherapeutic agents. Evidence suggests that the ERCC1 C118T SNP could affect mRNA and protein expression of ERCC1, therefore altering sensitivity to platinum-based therapeutics [5], [6]. Meta-analysis suggests that certain ERCC1 SNPs may also be predictive of cancer susceptibility [7], further highlighting potential importance of this genetic alteration in cancer detection and therapeutics.

Although the link between platinum-based chemotherapeutics and ERCC1 is debatable, the predictive aspect of assessing ERCC1 polymorphisms is attractive for assessing risk of cancer development in esophageal cancer due to the high latency of detection and resulting poor outcomes in this disease. Interestingly, no studies have investigated the relationship between ERCC1 and histopathological and clinical characteristics of esophageal cancer. Combined treatment of chemotherapy and radiation is considered one of the most useful therapeutic paradigms for esophageal squamous cell carcinoma (ESCC) [8], [9], which potentiates a connection between genetic factors like ERCC1 SNP and individual treatment outcome. The present study aimed to investigate the relationship between ERCC1 SNP and prognostic indication, histological and clinical outcomes in patients undergoing treatment for squamous cell esophageal carcinoma.

## Materials and Methods

### Study population

Written informed consent was obtained from all patients prior to the study via an institutional patient consent form. The study and this consent procedure were approved by the Ethics Committee of the First Hospital of Quanzhou Affiliated to Fujian Medical University. One hundred eight patients with surgically resectable thoracic esophageal squamous cell carcinoma (ESCC) treated in our hospital between January 2011 and October 2012 were enrolled in the present study. All patients were residents of Quanzhou, in southeastern Fujian Province, China (Figure S1). Informed consent was obtained from all patients prior to the study. The study was approved by the Ethics Committee of the First Hospital of Quanzhou Affiliated to Fujian Medical University. Patients received curative esophagectomy, and lymph node dissection that involved two-field lymphadenectomy in 94 patients (87%) and three-field lymphadenectomy in14 patients (13%). T4 tumors were resectable but had invaded lung, pleura, or recurrent nerve tissue. None of the study participants received neoadjuvant therapy. Resected specimens were collected and classified according to the International Union Against Cancer tumor–node– metastasis (TNM) classification system (7th ed.) [10]. Every 3 months following discharge, all patients were followed up with radiographic and computed tomography (CT) examination. At 6 months, ultrasound was utilized, and 6 to 12 months included endoscopic examination to assess disease status. Median follow up time range was 3–136 months. Patient clinicopathological characteristics are summarized in Table 1.

**Table 1:** Patient characteristics.

Characteristics	N ( = 108)
Gender(male/female)	87/21
Mean Age (yrs)	58.4±9.5
Tumor location	
Upper/middle/lower	5/62/41
TNM Classification	
T1/T2/T3/T4	11/21/46/30
N0/N1/N2/N3	58/33/13/4
Grade I/II/III	8/80/20
Adjuvant therapy	
None/chemo/radiation/both	67/31/7/3

T: Tumor stage; N: Nodal spread.

### Adjuvant platinum and radiotherapy

Thirty one patients received adjuvant intravenous platinum-based chemotherapy (75 mg/m2 cisplatin, 3–5 days every 3 weeks) and seven received radiotherapy (2 Gy, 5d/week for 4 weeks) to the mediastinum and neck. Three patients received post-surgery adjuvant chemo- and radiotherapy according to these treatment parameters. Sixty-seven patients received no adjuvant therapy in addition to surgical tumor resection.

### Immunohistochemical analysis of nuclear ERCC1

Immunohistochemistry (IHC) was performed on formalin-fixed tissue embedded in paraffin, and sectioned at 3 um thickness and mounted on positively charged slides. Tissue sections were deparaffinized in xylene, hydrated in descending alcohols, and treated with 3% H_2_O_2_ to quench endogenous peroxidase. Next, the tissue was treated with EDTA buffer (pH 9.0) and heated in a 98°C water bath for 30 min for antigen retrieval and nuclear access of the antibody. Sections were then blocked for non-specific binding, and incubated with mouse monoclonal ERCC1 (clone 8F1, Neomarkers, Fremont, California, USA; 1∶100 dilution) in blocking solution overnight at 4°C. The following day, visualization of ERCC1antibody labeling was achieved through use of the Envision Detections System (DAKO, Glostrup, Denmark) followed by 3, 3r-diaminobenzidine tetrahydrochloride (DAB Plus; DAKO) treatment. Hematoxylin counterstaining was also performed. For a positive control, normal human tonsil tissue was treated under the same conditions, and primary antibody exclusion was performed as a negative control.

**Figure 1:**
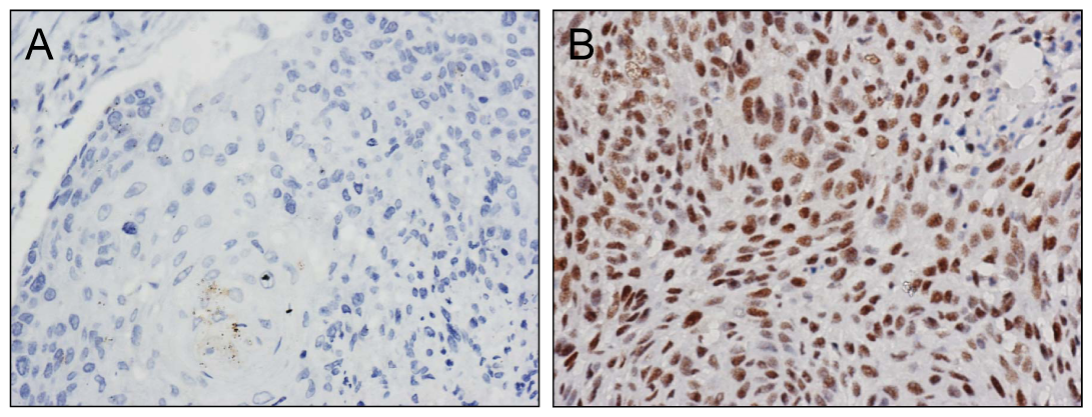
Representative immunohistochemical staining for ERCC1 in esophageal squamous cell carcinoma. Such staining was used for classification of nuclear staining intensity and assignment of a corresponding H-score used in further analyses in the study. A) ERCC1-negative esophageal squamous cell carcinoma tissue (H-score  = 0); B) High nuclear expression of ERCC1 in esophageal tumor cells with labeling intensity scored as 3 (H-score  = 3) (400×).

An experienced pathologist blinded to patient treatment conditions examined the labeling and semi-quantified ERCC1 expression using the H-score system described previously by Al Haddad et al. [11]. According to this system, ERCC1 staining intensity in tumor cell nuclei was graded from 0 to 3, with 0 indicating no staining and 3 designating high expression labeling (Figure 1). The extent of staining was classified as follows: 0 =  no nuclear expression; 0.1 = 1 to 9% labeled nuclei; 0.5 = 10 to 49% positive nuclei; and 1.0 = ≥50% ERCC1 positive nuclei. The value given for staining intensity was multiplied with the staining extent value to obtain the semi-quantitative H score. ERCC1^+^ cells were distinguished from ERCC1^−^ cells by the median value of all H scores (2.0). When >50% tissue was lost or no tumor cells were observed, samples were deemed uninterpretable.

### PCR polymorphism analysis

**Figure 2:**
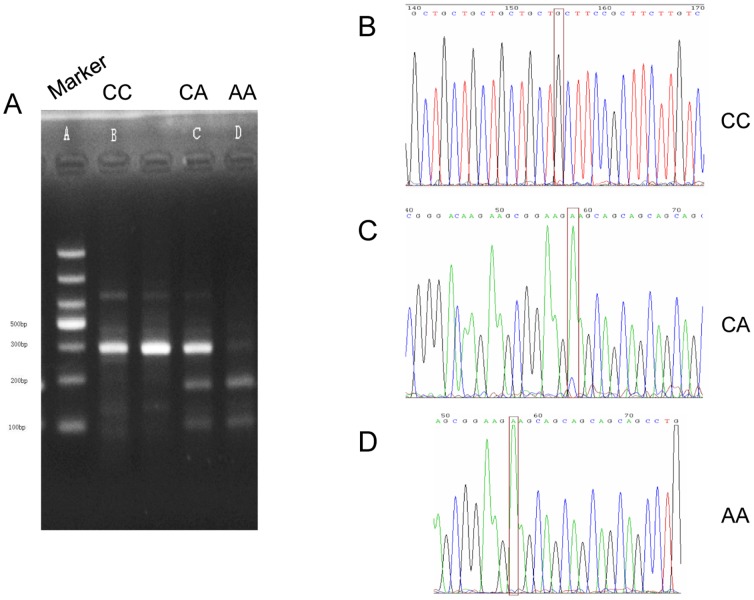
Genotype analysis for ERCC1 SNP C8092A. A) Post-digestion PCR of alleles CC, CA, and AA (left to right). B) Sequence analysis of CC genotype. C) Sequence analysis of CA heterozygous genotype. D) Sequence analysis for the AA genotype.

**Figure 3:**
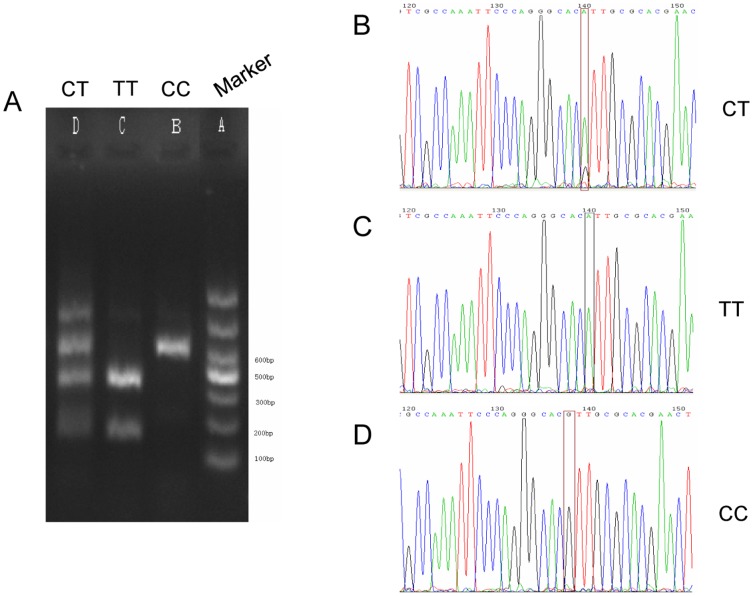
Genotype analysis for ERCC1 SNP C118T. A) Post-digestion PCR of alleles CT, TT, and CC (left to right). B) Heterozygous sequence analysis of CT genotype. C) Sequence analysis of TT genotype. D) Sequence analysis of CC genotype.

DNA was extracted from frozen resection samples collected from 40 patients diagnosed with ESCC using a genomic extraction kit for PCR reaction. Primer sequences utilized for polymorphism analysis of the rs11615 gene locus (BsrD I restriction site): Upstream primer: 5′-CATGCCCAGAGGCTTCTCATA-3′, Downstream primer: 5′-AGGACCACAGGACACGCAGAC-3′; for ERCCl gene 8092 (MboII restriction sites) polymorphism analysis: Upstream primer: 5′- TGCCAGAGACAGTGCCCCAAG-3′, Downstream primer: 5′-AGCTGCCAAGGAAACCCCCAG-3′. The total PCR reaction mix volume was 25 µl and annealing temperatures were 60°C; PCR products were digested separately, and the ERCCl gene was amplified by PCR and restriction enzyme digestion system reaction conditions were observed in Annex. The reaction mixture and conditions for each polymorphism analysis are shown in Table S1. The amplified fragment length of the ERCCl-rs11615 allele was 542 bp. The PCR products were digested by restriction enzyme BsrD I and the ERCCl-rs11615C → T polymorphism was determined. The analyzed genotype information obtained was as follows: wild-type homozygous C/C (542 bp), heterozygous C/T (542 bp, 175 bp, 367 bp), homozygous mutant T/T 175 bp, 367 bp). After the 285 bp ERCCl-8092 allele fragment was amplified, the product was digested by MboII restriction enzyme and polymorphisms were determined for ERCCl-8092C → A: wild-type homozygous C/C (285 bp), heterozygous type C/A (285 bp, 104 bp, 181 bp), homozygous mutant A/A (104 bp, 181 bp). In each of the three genotypes, three randomly selected PCR product samples were sequenced (Shanghai Baoshang Co.). The sequencing results were consistent with those obtained following digestion and PCR (Figures 2 and 3).

### Determination of genotypic Hardy-Weinberg equilibrium

Assessment of whether allelic variations existed in Hardy-Weinberg equilibrium was calculated according to software and methods previously described [12]. In brief, allelic frequencies of C8092A and C118T polymorphisms were entered into a software calculator based on the equation, p2+2pq+q2 = 1, where p represents the major allele, q the minor allele, and pq the heterozygous condition. A *P* value <0.05 would suggest the allelic distribution does not fit classic Hardy-Weinberg equilibrium.

### Statistical analysis

Statistical comparison of ERCC1 expression with clinical and pathological features was performed using a non-parametric Mann-Whitney U test, whereas genotypic relationship to tumor ERCC1 expression was determined using a chi-square test. Overall and progression-free (PFS) survival curves were calculated by the Kaplan–Meier method, and the differences were assessed by the log-rank test. SPSS software (ver. 9.2, StataCorp, College Station, TX, USA) was used for statistical analysis with significance determined when *P*<0.05.

## Results

### Adjuvant therapy and esophageal squamous cell carcinoma survival

**Figure 4:**
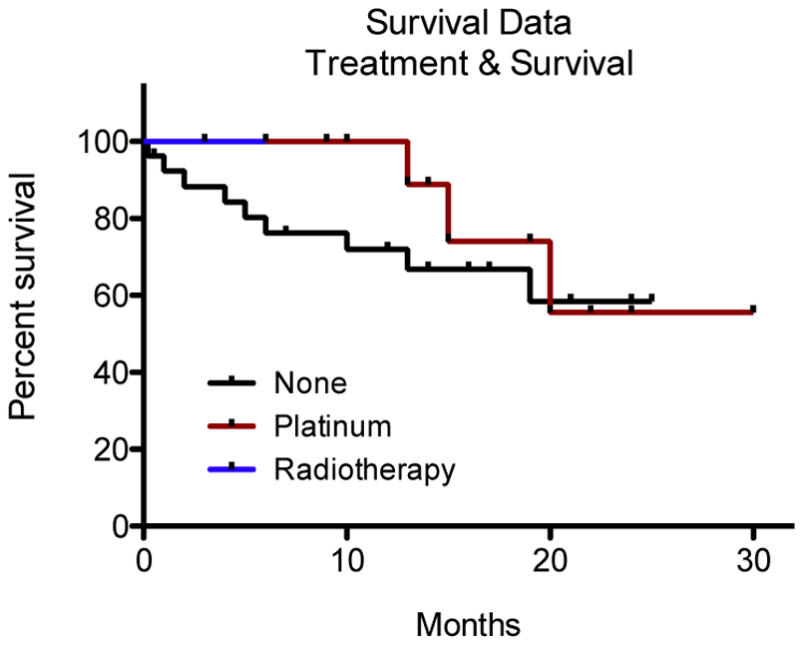
Adjuvant treatment did not significantly influence patient survival probability. Patient survival probability was not significantly influenced by platinum-based chemotherapy or radiation over no adjuvant therapy. Log-rank (Mantel-Cox) Test; Chi-square  = 2.0; p = 0.56.

Of the 108 patients that underwent surgical tumor resection, 67 (62%) received no adjuvant therapy, 31 (28.7%) received platinum-based chemotherapy, and 7 (6.5%) had adjuvant radiotherapy. Three patients (2.8%) underwent both chemo- and radiotherapy following surgical resection. Kaplan-Meier survival curves are shown in Figure 4. The results of Log-rank (Mantel-Cox) analysis of the survival data showed no significant association between use or type of adjuvant therapy and survival (*P* = 0.56). Log-rank test for trend revealed no significant trend concerning treatment effect on survival (*P* = 0.40).

### Association between ERCC1 histological analysis and patient survival

**Figure 5:**
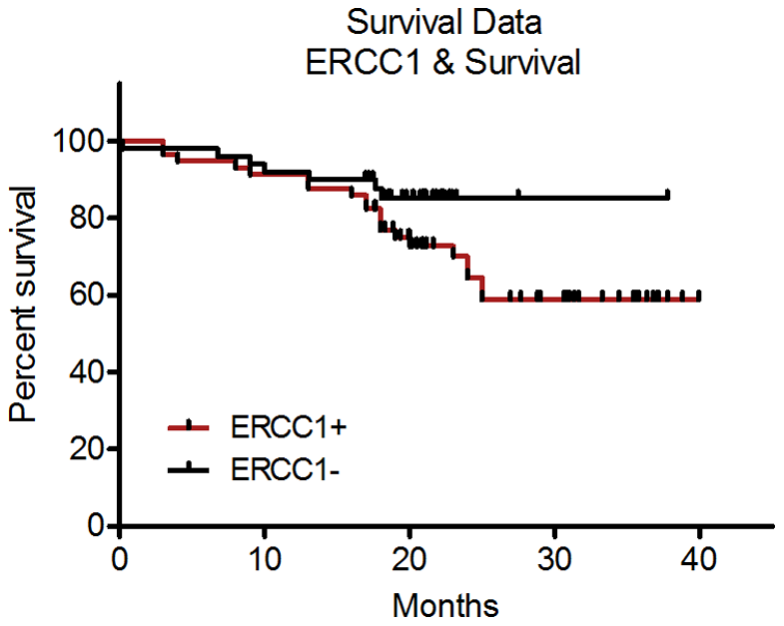
Patient survival was not significantly associated with ERCC1 nuclear expression H-score. There was no significant relationship between high or low intensity ERCC1 nuclear immunohistochemical labeling designated by H-score cutoff of 2.0 and patient survival. Chi- square  = 0.27; p = 0.61. Median patient survival (months: ERCC1+, 23.8; ERCC1-, 20.3, 95% CI of ratio: 0.8341 to 4.227.

Tumor samples from fifty-six patients (52%) were identified as ERCC1-positive with an H-score ≥2.0. Log-rank (Mantel-Cox) analysis identified no significant link between histologically defined ERCC1 status and overall and progression-free patient survival through the course of the study (*P* = 0.13). Kaplan-Meier curves are shown in Figure 5.

### ERCC1 expression and esophageal tumor location

Genetic and statistical analysis showed that H-score designated ERCC^+^ (n = 56) and ERCC^−^ (n = 52) status of enrolled patients had no statistically significant association with tumor location within the esophagus identified at the time of surgery (*P* = 0.94) (Table 2).

**Table 2:** Clinicopathological patient characteristics according to ERCC1 status.

	ERCC1 negative	ERCC1 positive	*P* value
	H score<2.3 (*n*)	H score≥2.3 (*n*)	
Tumor location			0.94
Upper	4	1	
Middle	27	35	
Lower	21	20	
Tumor grade			0.80
1	5	3	
2	37	43	
3	10	10	
Tumor size			0.08
T1	7	4	
T2	13	8	
T3	20	26	
T4	12	18	
Lymph node status			0.33
N0	31	27	
N1	13	20	
N2	6	7	
N3	2	2	
C8092A			0.56
CC	7	12	
AA	27	18	
CA	18	26	
C118T			0.37
CC	32	33	
TT	7	6	
CT	13	17	

ERCC1: excision repair cross-complementation 1 enzyme, * *P* value is statistically significant.

### ERCC1 and tumor grade

In individuals with tumors identified as ERCC1^−^, 9.6% of tumors were Grade I, while the majority was classified as Grade II (71.2.%), or III (19.2%). 17.9% of ERCC1^+^ tumors were classified as Grade III, 76.8% Grade II, and 5.4% Grade I. Based on differences in the distribution of tumor gradation by nuclear ERCC1-labeling status, statistical analysis revealed no significant association between these variables (*P* = 0.80) (Table 2).

### ERCC1 and lymph node tumorigenesis

The vast majority of nodes evaluated in regions adjacent the primary tumor location in all patients were assigned values of either 0 or 1 (ERCC1^−^  = 81.5%, ERCC1^+^  = 83.9%) suggesting limited nodal cancer spread and development. Correspondingly, statistical analysis showed no significant relationship between nuclear ERCC1 status and node value assignment (*P* = 0.94) (Table 2).

### Allelic discrimination and ERCC1 histological characterization

For ERCC1 polymorphism C8092A, approximately 40% of patients were homozygous for the major allele (CC = 40.7%) or heterozygous (CA = 41.7%) while only 17.6% were homozygous for the recessive allele (AA). Hardy-Weinberg calculation showed these values to be at equilibrium (*P* = 0.21). For tumors indicated ERCC1^+^ with an H–score ≥2.0, 46% of patient samples were homozygous for the major allele (CC) for ERCC1-C8092A, 21% for the minor allele (AA) and 33% were heterozygous (C/A). For ERCC1^−^ samples, 33% were homozygous CC, 13% homozygous AA and 54% C/A. No significant relationship was identified between ERCC1 staining and allelic distribution of SNP C8092A (*P* = 0.56) (Table 2).

Concerning polymorphism C118T, the allelic distribution of all patient samples was not determined to be at Hardy-Weinberg equilibrium (*P* = 0.008). ERCC1^+^ samples showed an allelic distribution with most being homozygous for the major allele (CC = 59%), a trend also observed from ERCC1^−^ tumor samples (CC = 59.2%) (Table 2). ERCC1-positive to negative comparison for minor allele homozygotes (TT) was 10.7% to 11.1%, respectively. Of ERCC1^+^ samples, 30.4% were heterozygous (C/T) compared to 24.1% of ERCC1^−^ specimens. Ultimately, statistical analysis revealed no significant association between ERCC1 status and allelic distribution for polymorphism C118T (*P* = 0.37) (Table 2).

**Figure 6:**
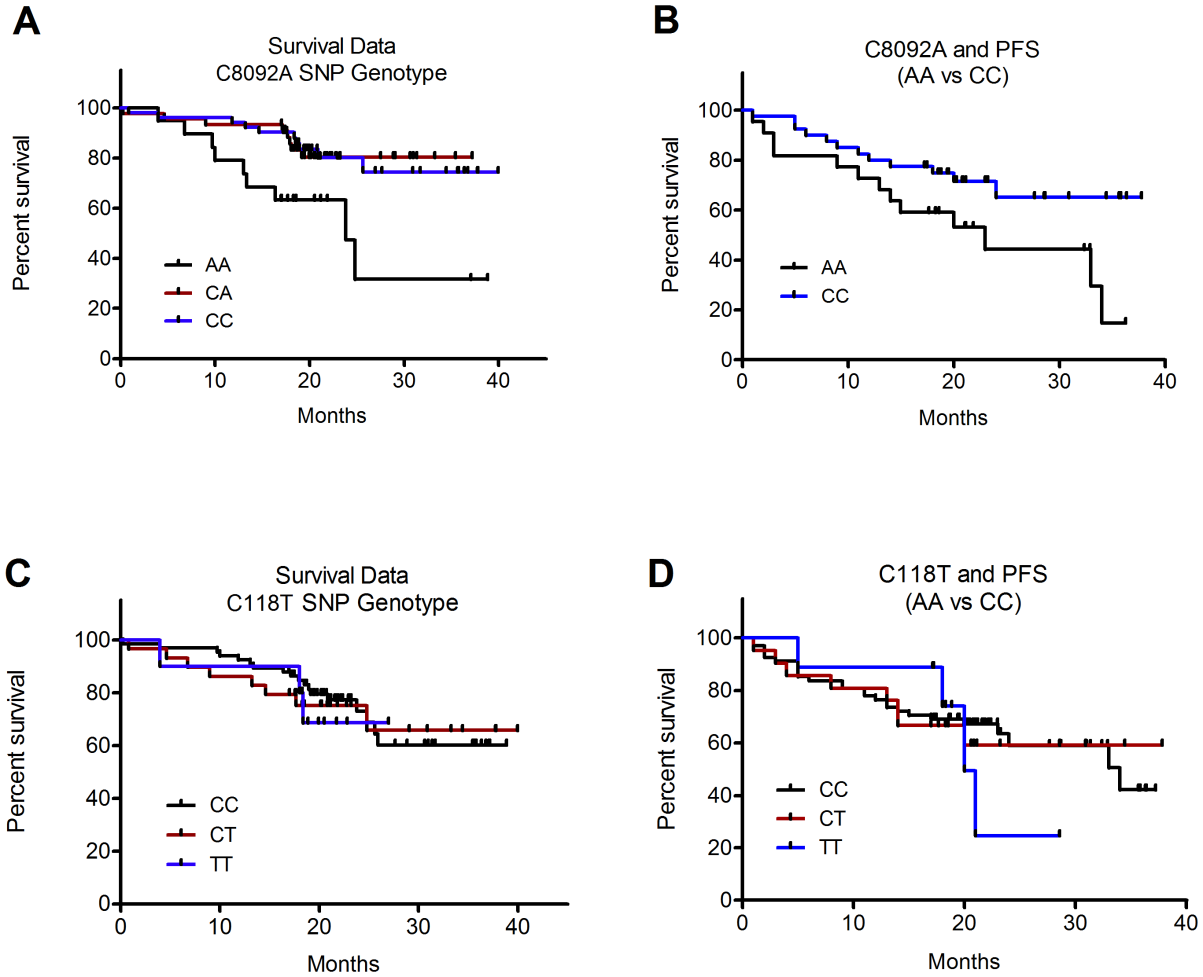
Association of ERCC1 polymorphism allelic variation on patient survival. A) ERCC1 polymorphism C8092A was significantly associated with overall patient survival (p = 0.01). Presence of the mutant allele, especially for the homozygous patients for this allele (AA), was associated with decreased overall and progression-free survival (PFS) compared to homozygous wild-type patients (CC) (p = 0.007 & 0.04, respectively). No significant relationship between polymorphism genotype and C) overall patient survival for C118T (p = 0.78) or D) PFS was observed (*P* = 0.93).

Unlike ERCC1 histological classification, genotype analysis for the C8092A polymorphism exhibited a significant association with patient outcomes and overall and progression-free (PFS) survival. Specifically, log-rank analysis identified a significant relationship between variation of alleles identified in patient samples for C8092A (chi-square  = 9.1, *P* = 0.01). Concerning PFS, no significant association was determined when analyzing all three allelic variations (chi-square  = 4.2, *P* = 0.12), although a significant trend was detected (chi-square  = 4.1, *P* = 0.04). When on the homozygous dominant wild-type (CC) vs. the mutant (AA) were assessed, a significant reduction in survival was determined in those with the AA genotype (chi-square  = 4.3, *P* = 0.04). No significant association on overall survival was observed for C118T (chi-square  = 0.08, *P* = 0.96). There was also no significant trend observed for SNP allele variations of C118T concerning association with overall survival (*P* = 0.78) or PFS (chi-square  = 0.15, *P* = 0.93). Kaplan-Meier survival curves for C8092A are illustrated in Figure 6A & B, and C118T in Figure 6C & D.

## Discussion

Platinum-based chemotherapeutics target and damage DNA, advancing cell death of affected tumorigenic, as well as normal cells. Cells produce DNA repair complexes and enzymes to mend nucleic acid damage caused by platinum agents and impart resistance against such treatments [13]. Repair proteins, such as ERCC1 are therefore important for cancer cells resistance to platinum and other DNA-targeting or altering therapeutics. Polymorphisms in ERCC1 gene affect mRNA expression and have received much attention as potential predictors of cisplatin and platinum adjuvant therapy outcome, and patient prognosis in lung cancer [14]–[16], melanoma [17], bladder cancer [18], [19], in addition to esophageal cancer [1], [20]–[23].

In the present study, we investigated the association between tumor ERCC1 expression and molecular, histological, and clinicopathological characteristics of a cohort of multi-stage patients treated and not treated with adjuvant platinum-based chemotherapy or radiotherapy following curative resection surgery. Our findings are interesting as some of the data counter recent reports of the predictive ability of ERCC1 expression and treatment outcome and survival of patients with esophageal cancer [21]. Also, our results do not support semi-quantitative association between tumor ERCC1 nuclear protein expression and clinical and pathological outcomes in the patient cohort in this study. Warnecke-Eberz et al. showed that ERCC1 mRNA expression was prognostic for histopathologic response to neoadjuvant radiochemotherapy involving cisplatin in patients with locally advanced esophageal cancer in combination with surgical tumor removal [24]. A study by Brabender and colleagues supported this result [20]. Tanaka et al. demonstrated reduced ERCC1 mRNA correlated with improved platinum-based chemoradiation therapy response in patients with ESCC, and suggested that histological assessment of tumor ERCC1 may predict individual responses to the adjuvant treatment paradigm [25]. ERCC1 nuclear protein expression is also suggested to be predictive of patient response to platinum-based chemotherapy [26]. Our study does not corroborate these results based on our blinded categorization of high- and low-intensity ERCC1 labeling of resected tumor tissue and analysis of association between ERCC1 expression and patient outcomes. Also, we saw no treatment-specific effects on overall patient survival, suggesting our patient cohort may not be sensitive to effects of adjuvant platinum-based chemotherapy following curative tumor dissection. As neither tumor ERCC1 expression nor adjuvant treatment was significantly related with patient outcome, it seems unlikely these two variables interacted. Of the analyses we did perform, we found no association between tumor location, status, or nodal spread on H-score defined tumor expression of ERCC1 protein supporting this conclusion.

Contrary to what we observed for ERCC1 H-score classification and adjuvant therapy, some polymorphic ERCC1 influence on overall patient survival was identified. Two commonly studied ERCC1 SNPs occur at loci C8092A and C118T, and both have been suggested to predict treatment response or patient outcome in esophageal cancer [22], [23], [27]. Of the two, the C8092A SNP showed a significant association with overall and progression-free patient survival. It is documented that polymorphisms that disrupt DNA base repair increase risk of ESCC [28], [29]. In our study, the C → A mutation at ERCC1 residue 8092 appears to diminish overall survival and PFS, which is reasonable if this mutation is associated with increased risk of ESCC development. However, as all patients in our study had some degree of ESCC, this SNP may provide a negative prognostic marker for certain patient populations already diagnosed with ESCC.

Our location in Fujian province in China could have provided a unique insight into population specific determinants of ESCC progression, treatment effects, and patient survival. Upon review of the literature, many recent studies investigating polymorphic variations in ERCC1 and the predictive potential for patient and platinum-based treatment effects take place in Europe or the United States where ESCC is far less common than esophageal adenocarcinoma. A unique aspect of our study is that the patient population did not receive neoadjuvant therapy before surgery, as is commonly included in other similar analyses. The patients in the present study received treatment following surgery, which could considerably influence outcome parameters and may not be reflected in the pre-treatment analysis of surgically-resected tumor samples.

As previously described, squamous cell esophageal cancer is conversely more common in China. One recent study explored ERCC1-C8092A polymorphic predictive value of ESCC susceptibility in North Xinjiang, China [30]. This particular study observed no increased risk for development of ESCC with this SNP, and thus, complications stemming from the disease. In the present study however, an association between this loci and increased risk of ESCC-related mortality was observed. Here, when all three allelic combinations were statistically compared, those with the AA genotype showed a trend in reduced overall survival, and when two homozygous allelic combinations (AA vs. CC) were compared there was a clear significant influence on survival, with patients homozygous for the mutant allele (AA) exhibiting reduced overall survival than those homozygous for the dominant allele, CC (*P* = 0.07, Hazard Ratio  = 4.496, 95%CI  = 1.507 to 13.42). Interestingly, one recent study showed that ERCC1-C8092A SNP also was linked to increased risk of colorectal cancer in a Chinese population [31]. Similar to our findings, the recessive mutant allele (A) in their study was directly related to overall patient survival in both the homozygous mutant (AA) and heterozygous (CA) genotypes. When we analyzed association between ERCC1 expression status and those with the AA/CA vs. CC genotypes, still no significant connection was found (*P* = 0.25). As such, the C → A mutation decreases overall survival in our study patient population, but actual ERCC1 expression level within analyzed tumor tissue was not associated with any genotypic variation at this loci. Despite this connection between ERCC1-C8092A SNP and patient survival, assessment of the full implications of these findings in the context of our study is complicated. There may be regional differences, even within China that contribute to one population's risk of developing or dying from cancer, and such differences or other influences not addressed in the present study may affect risk of different forms of gastric cancer. A more detailed study examining the potential interacting effects of other SNPs or adjustments for confounding lifestyle factors such as smoking or comorbidities may yield more definitive interpretation and discussion.

One notable outcome of the present study showed was that no significant connection was observed between ERCC1 expression and clinicopatholgic progression of ESCC in the study population. Our analyses also showed no significant association between ERCC1 tumor expression and prognosis of patient outcome. These results are supported by findings in a recent study by Fareed et al. that showed no association between histological tumor nuclear ERCC1 expression and patient survival, tumor stage, nodal invasion, or vascular involvement in gastroesophageal adenocarcinoma [26]. Using a similar approach, one recent retrospective study analyzed resected tumor tissue immunohistochemistry for ERCC1 to determine associations between tumor expression of the protein and tumor characteristics or survival in patients with non-small cell lung cancer [32]. This study supported our findings that ERCC1-tumor expression had no bearing for predicting patient survival or tumor recurrence regardless of adjuvant chemotherapy. da Costa Miranda et al. also found no predictive benefit for tumor ERCC1 immunohistochemistry on patient survival or tumor response [33].

On the other hand, Joshi et al. found a link between elevated gene expression of ERCC1 and decreased survival in patients with esophageal cancer, despite increased survival in response to multimodal treatment involving cisplatin and 5-fluorouracil (5-FU) [34]. It must be noted that the association between ERCC1 and survival was an observed trend, and not statistically significant. This groups' study enrolled nearly double the patients than the present study, which complicates the comparison of our results and those by Joshi and colleagues. Also, their report states the patient cohort was a mixture of squamous cell and adenocarcinoma, further clouding any potential comparison. In line with these findings, Yang and Xian performed a meta-analysis on clinical studies of various ERCC1 SNPs including C118T in non-small cell lung cancer and found overall reduced survival in ERCC1^+^ patients, as well as reduced sensitivity to platinum-based chemotherapy [35].

Two novel aspects of our study are that we 1) examined tumor and patient survival characteristics based on histological presence or absence of ERCC1 in the nuclei of tumor cells, and 2) our patient cohort consisted of individuals from Quanzhou, Fujian Province, in southeastern China, an understudied region concerning genetic variation association with ESCC and treatment response outcomes (Figure S1). To our knowledge this is the first clinical analysis of ERCC1 expression and polymorphic variation in esophageal cancer patients in Fujian Province. Only two other studies from this province have involved examination of ERCC1 polymorphisms and cancer, however these were focused on colorectal [36] and liver cancer [37]. Our sample size and the study itself was not large enough to make firm and specific claims based on the extent of data analysis performed here; rather, potential explanations for observed outcomes opens doors for future investigation. Our findings corroborate other reports showing minimal predictive ability for specific ERCC1 expression in predicting patient treatment response and survival. Few studies have targeted tumor cell-specific expression of ERCC1 as a potential correlate for the progression of squamous cell carcinoma of the esophagus, and our findings yield interesting potential associations between ERCC1 SNP C8092A and survival of patients with this disease. This might prove valuable for assessment of other allelic variations or SNPs of ERCC1 in regards to treatment response and survival outcomes in this form of the disease.

Also, the patient population in the present study is unique and has not been previously characterized concerning patient outcome from ESCC, surgical resection, or adjuvant therapy. This is the first study to investigate this regional population of Chinese with ESCC, and linked ERCC1 polymorphism to esophageal carcinoma in these patients that impacted overall survival. Otherwise, we found no statistical relationship between ERCC1 nuclear protein expression or platinum/radiotherapy adjuvant treatment and patient survival following surgical tumor resection in ESCC. A larger patient population and further consideration of population-specific genotypic factors will allow validation of the present study and improve the statistical power of future research concerning ERCC1 expression and polymorphic variation on patient and treatment outcome in ESCC.

## Supporting Information

Figure S1 **Geographic information on study location and patient population.** A & B) The present study took place and enrolled patients from Fujian Province in southern China. C) Specifically, the study focused on residents of the city of Quanzhou, located in the southeastern region of Fujian Province.
(TIF)

Table S1 **PCR and restriction enzyme digestion system and reaction conditions for ERCCl gene amplification.**
(DOCX)
