# Correction: ERCC1 Single Nucleotide Polymorphism C8092A, but Not Its Expression Is Associated with Survival of Esophageal Squamous Cell Carcinoma Patients from Fujian Province, China

**DOI:** 10.1371/journal.pone.0115487

**Published:** 2014-12-05

**Authors:** The PLOS ONE Staff

In Table 2, the heading indicates an H score cutoff of 2.3. The correct cutoff is 2.0. Please see the corrected Table 2 below.

**Table 2:** Clinicopathological patient characteristics according to ERCC1 status.

	ERCC1 negative	ERCC1 positive	*P* value
	H score<2.0 (*n*)	H score≥2.0 (*n*)	
Tumor location			0.94
Upper	4	1	
Middle	27	35	
Lower	21	20	
Tumor grade			0.80
1	5	3	
2	37	43	
3	10	10	
Tumor size			0.08
T1	7	4	
T2	13	8	
T3	20	26	
T4	12	18	
Lymph node status			0.33
N0	31	27	
N1	13	20	
N2	6	7	
N3	2	2	
C8092A			0.56
CC	7	12	
AA	27	18	
CA	18	26	
C118T			0.37
CC	32	33	
TT	7	6	
CT	13	17	

ERCC1: excision repair cross-complementation 1 enzyme, * *P* value is statistically significant.
