# Supplementary figures and images for: ERCC1 Single Nucleotide Polymorphism C8092A, but Not Its Expression Is Associated with Survival of Esophageal Squamous Cell Carcinoma Patients from Fujian Province, China
Source: PLoS One. 2014 Sep 5;9(9):e106600. doi: 10.1371/journal.pone.0106600 (PMC4156356; doi:10.1371/journal.pone.0106600)

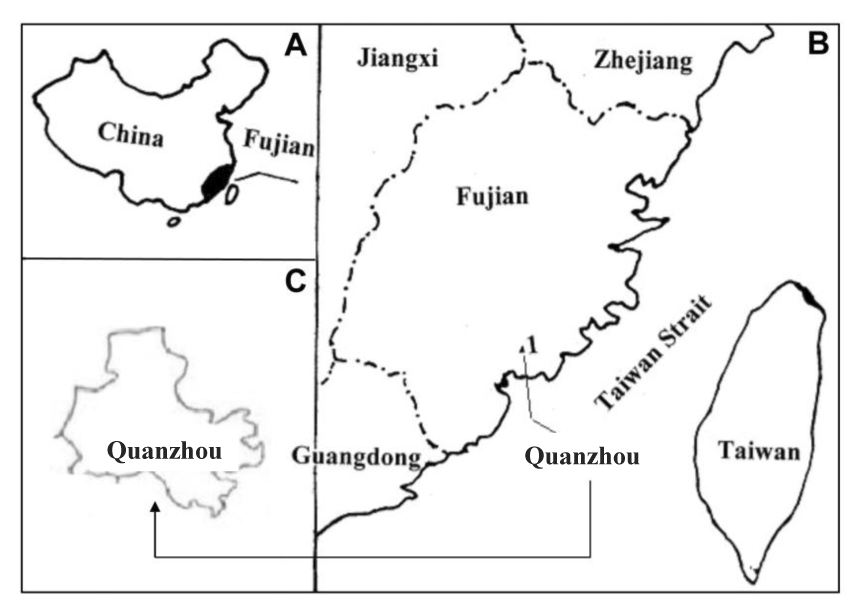

Supplement: Figure S1 — Geographic information on study location and patient population. A & B) The present study took place and enrolled patients from Fujian Province in southern China. C) Specifically, the study focused on residents of the city of Quanzhou, located in the southeastern region of Fujian Province. (TIF) [file pone.0106600.s001.tif]
