# Supplementary material for: ERCC1 Single Nucleotide Polymorphism C8092A, but Not Its Expression Is Associated with Survival of Esophageal Squamous Cell Carcinoma Patients from Fujian Province, China
Source: PLoS One. 2014 Sep 5;9(9):e106600. doi: 10.1371/journal.pone.0106600 (PMC4156356; doi:10.1371/journal.pone.0106600)
Supplement: Table S1 — PCR and restriction enzyme digestion system and reaction conditions for ERCCl gene amplification. (DOCX) [file pone.0106600.s002.docx]

Table S1: PCR and restriction enzyme digestion system and reaction conditions for ERCCl gene amplification

| **Reagents** | **Digestion system** | |
| --- | --- | --- |
|  | ERCCl-118 | ERCCl-8092 |
| ddH_2_O | 7.3 µl | 7.5 µl |
| Buffers | 2 µl | 2 µl |
| BsrD I | 0.5 µl | - |
| BsA | 0.2 µl | - |
| MboII | - | 0.5 µl |
| PCR product | 10 µl | 10 µl |
| Reaction conditions | 65 ^o^C bath (4 hrs) | 37 ^o^C bath (4 hrs) |
